# *TNFSF15* promoter polymorphisms increase the susceptibility to small cell lung cancer: a case-control study

**DOI:** 10.1186/s12881-019-0762-6

**Published:** 2019-02-08

**Authors:** Hui Gao, Zeren Niu, Zhi Zhang, Hongjiao Wu, Yuning Xie, Zhenbang Yang, Ang Li, Zhenxian Jia, Xuemei Zhang

**Affiliations:** 1 College of Life Science, North China University of Science and Technology, Tangshan, 063210 China; 2 School of Public Health, North China University of Science and Technology, Tangshan, 063210 China; 3 Affliated Tangshan Gongren Hospital, North China University of Science and Technology, Tangshan, 063000 China; 4 School of Basic Medical Sciences, North China University of Science and Technology, Tangshan, 063210 China

**Keywords:** *TNFSF15*, Lung cancer, Single nucleotide polymorphism, Cancer susceptibility

## Abstract

**Background:**

Tumor necrosis factor superfamily member 15 (TNFSF15) is closely related to tumorigenesis and development. This study aimed to investigate the correlations between *TNFSF15* polymorphisms and genetic susceptibility to lung cancer.

**Methods:**

This case-control study included 209 small cell lung cancer patients (SCLC), 340 non- small cell lung cancer patients (NSCLC) and 460 health controls. *TNFSF15*–638 A > G and − 358 T > C polymorphisms were genotyped by polymerase chain reaction-restrictive fragment length polymorphism (PCR-RFLP) analysis. Odds ratio (OR) and 95% confidence interval (95% CI) were estimated by unconditional logistic regression.

**Results:**

Our results showed that subjects carrying the *TNFSF15–*638GG genotype or -358CC genotype were more likely to develop SCLC (−638GG, *OR* = 1.84, 95%*CI* = 1.13–2.99; -358CC, *OR* = 2.44, 95%*CI* = 1.46–4.06), but not NSCLC (*P* > 0.05). In stratified analysis, −638GG genotype was related to SCLC among males (*OR* = 1.95, 95%*CI* = 1.09–3.45, *P* = 0.023) and older patients (*OR* = 2.93, 95%*CI* = 1.44–8.68, *P* = 0.006). However, -358CC genotype was associated with SCLC among females (*OR* = 8.42, 95%*CI* = 2.22–31.89, *P* = 0.002) and older subjects with *OR* (95%*CI*) of 11.04 (3.57–34.15) (*P* < 0.001). Moreover, *TNFSF15* -358CC was linked with a higher risk of SCLC among non-smokers (*OR* = 2.54, 95%*CI* = 1.20–5.35, *P* = 0.015) but not among smokers (*OR* = 1.88, 95%*CI* = 0.92–3.84, *P* = 0.086).

**Conclusion:**

These findings highlight the importance of *TNFSF15* polymorphisms in the development of SCLC.

## Background

Lung cancer is one of the most common malignant tumors worldwide and the first leading cause of cancer-related mortality in China [1, 2]. According to World Health Organization, lung cancer is divided into two main types: non-small cell lung cancer (NSCLC) and small cell lung cancer (SCLC), of which NSCLC accounts for almost 85% of lung cancer cases [3]. Epidemiological studies have identified several risk factors for lung cancer, such as tobacco smoking, atmospheric pollution and occupational environment challenge [4]. However, many individuals who have been exposed to these risk factors do not get lung cancer during lifetime, so genetic factor is likely play an important role.

The initiation and progression of cancer are closely linked to inflammation and angiogenesis [5, 6]. As one of potent mediators of inflammation, the tumor necrosis factor (TNF) family plays an important role in the process of immunoregulation and further contributes to cancer development [7]. Tumor necrosis factor superfamily 15 (*TNFSF15*), also known as vascular endothelial growth inhibitor (*VEGI*), belongs to the *TNF* ligand family, which negatively regulates angiogenesis [8]. By stimulating T cell, TNFSF15 is involved in the modulation of inflammation [9, 10]. Studies have shown that over-expression of TNFSF15 inhibits tumor growth in various cancers whereas reduced expression of TNFSF15 is associated with poor prognosis in cancer patients [11–15].

Single nucleotide polymorphisms (SNPs) in regulatory region of a gene can influence the gene expression and further contribute to the development of various cancers [16–19]. In our previous study, we identified two SNPs (−638A > G and -358 T > C) in the *TNFSF15* promoter by direct sequencing, and found that -358 T > C variant changed the transcriptional activity of *TNFSF15* and was significantly associated with the susceptibility to gastric adenocarcinoma [20]. In the present study, we tested if these two variants in the *TNFSF15* promoter region contributed to the risk of developing lung cancer by performing a case-control study in a Chinese population.

## Methods

### Study population

This case-control study consisted of 209 SCLC patients, 340 NSCLC patients and 460 healthy controls (Table 1). The 549 cases were collected from Tangshan Gongren Hospital and Tangshan Renmin Hospital affiliated to North China University of Science and Technology in China from 2012 to 2016. None of the patients were treated with any radiotherapy or chemotherapy before blood sampling. All subjects were unrelated ethnic Han Chinese. Control individuals without a history of any cancer were recruited from the same region and frequency-matched to cases according to gender and age. This study was approved by Institutional Review Board of North China University of Science and Technology, and written informed consents were obtained from all participants of their own free will.

**Table 1:** Frequency distribution of select characteristics

Variables	NSCLC					SCLC				
	Case (*n* = 340)		Controls(*n* = 460)		*P* value^*^	Case (*n* = 209)		Controls(n = 460)		*P* value^*^
	No	(%)	No	(%)		No	(%)	No	(%)	
Gender					0.301					0.862
Male	237	69.7	336	73.0		154	73.7	336	73.0	
Female	103	30.3	124	27.0		55	26.3	124	27.0	
Age					0.139					0.151
<60	187	55.0	277	60.2		138	66.0	277	60.2	
≥ 60	153	45.0	183	39.8		71	34.0	183	39.8	
Range	27–84		18–84			30–92		18–84		
Median	58		56.2			55		56.2		
Smoking status					0.252					0.431
Non-smoker	187	55.0	233	50.7		99	47.8	233	50.7	
Smoker	153	45.0	227	49.3		110	52.2	227	49.3	
Pack year of smoking					< 0.001					< 0.001
<30	59	38.6	153	67.4		37	33.7	153	67.4	
≥ 30	94	61.4	74	32.6		73	66.4	74	32.6	

^*^Two-sided χ2 test

### *TNFSF15* genotyping

**Fig. 1:**
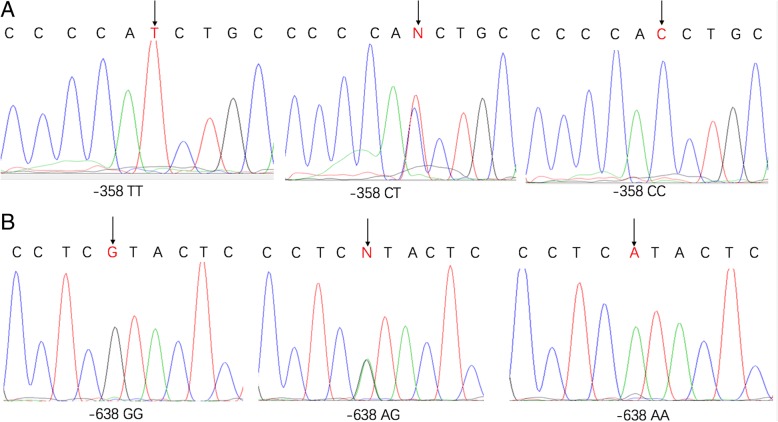
Sequence analyses of the *TNFSF15* promoter region reveal 2 SNPs located at nucleotides **a** − 638 A > G and **b** − 358 T > C. The arrows localize the base changes at the nucleotide positions

Genomic DNA was extracted from peripheral blood from all participants using TIANamp Blood DNA Kit (TIANGEN, Beijing, China), according to the manufacturer’s instructions. PCR-restriction fragment length polymorphism (PCR-RFLP) analysis were applied for *TNFSF15* genotyping. The PCR primer pairs for −638A > G (rs7848647) were 5′- AGT CAC CTC GAT CTG TGG CCTC-3′ and 5′-AAT CAC GGC TTG GAG TTG TAA CCTC-3′. The target DNA fragment containing − 358 T > C (rs6478109) was amplified with primer pairs, − 358 -PF (5′-AAA TGT GAT TTC CGT TTC CCCA-3′) and − 358 -PR (5′- AAT ATA CCT GTT CCC TGC ACTG -3′). Briefly, PCR was performed using 6 μL reaction mixture containing 10 ng DNA, 0.1 μM each primer, and 1 × Taq PCR StarMix with loading dye (Genstar, Beijing, China).The PCR thermal cycling condition consists of an initial denaturation step at 94 °C for 3 min, followed by 30 cycles of 94 °C for 40 s, 58 °C for 30 s and 72 °C for 15 s, and then a final extension step at 72 °C for 3 min. PCR products for *TNFSF15*–638A > G (114 bp) and -358 T > C (123 bp) were digested by Rsa *I* and Bcc *I* (New England BioLabs, Inc., Beverly, USA) and separated on 3% agarose gel. The genotypes revealed by PCR-RFLP were further confirmed by DNA sequencing (Fig. 1). To ensure the quality control, approximately10% of the samples were randomly selected for re-genotyping and all results were in 100% concordance.

### Statistical analysis

Quanto program was used to calculate the power of the sample size for this case-control study. The power estimation was performed, which indicated that our sample size is sufficient for the case-control study. Differences of basic characteristics in cases and control subjects were compared using the χ^2^ test. Pearson goodness-of-fit χ^2^ test was performed to test whether the distribution of genotypes in the control group was in accordance with Hardy-Weinberg Equilibrium (HWE). Odd ratios (ORs) and 95% Confidence interval (CI) were calculated by unconditional logistic regression model to evaluate the association of *TNFSF15* genetic variations with the susceptibility to lung cancer. The smoking status of pack-years was determined as an indicator of the cumulative cigarette dose level (pack-years cigarettes per day/20 × years smoked). Light and heavy smokers were categorized by using 30 as the cut-off point [21]. Older and younger subjects were sub-grouped by using 60 as the cut-off point (https://www.who.int). All statistical calculations were performed using SPSS version 23.0 (SPSS Inc., Chicago, IL).

## Results

### Demographic and clinical characteristics of lung cancer cases and controls

Demographic and clinical characteristics of lung cancer cases and controls are shown in Table 1. There was no significant difference in gender, age and smoking status between NSCLC or SCLC cases and healthy controls (*P* > 0.05). In terms of the amount of smoking, the significant difference was showed between lung cancer group and control group (*P* < 0.001 for NSCLC and *P* < 0.001 for SCLC). The proportion of heavy smokers in SCLC and NSCLC patients (66.4 and 61.4%) was significantly higher than that in healthy controls (32.6%).

### Association of *TNFSF15* variants with the risk of lung cancer

Tables 2 and  3 present the genotypes of *TNFSF15*–638A > G and -358 T > C variants in lung cancer patients and controls. Genotype distributions of *TNFSF15*–638A > G and -358 T > C in controls were conformed to be in the Hardy-Weinberg equilibrium (HWE) (*P* = 0.32 and *P* = 0.78, respectively). We used unconditional logistic regression to assess the association of *TNFSF15* SNPs with the risk of lung cancer. For the *TNFSF15*–638A > G polymorphism, we found that GG genotype carriers had a significantly elevated risk for developing SCLC (*OR* = 1.84, *95%CI* = 1.13–2.99), but not for developing NSCLC (*OR* = 1.11, *95%CI* = 0.74–1.67), in comparison to those with AA genotype. For the *TNFSF15* -358 T > C variant, our data showed that CC genotype and CT genotype were associated with a higher risk of SCLC (*OR* = 2.44, *95%CI* = 1.46–4.06; OR = 2.00, *95%CI* = 1.26–3.19) as compared to TT genotype. However, we did not find that the *TNFSF15* -358 T > C polymorphism was associated with the susceptibility to NSCLC with an *OR* *(95% CI)* of 1.45 (0.96–2.11) in CT carriers and an *OR (95% CI)* of 1.24 (0.86–1.76) in CC carriers, respectively.

**Table 2:** Genotype frequencies of *TNFSF15* polymorphisms and their association with SCLC

Genotype	Patients(n = 209)		Controls(n = 460)		OR (95%CI)^a^	*P* value
	No	(%)	No	(%)		
-638 A > G						
AA	39	18.7	122	26.5		
AG	112	53.5	240	52.2	1.46(0.95–2.23)	0.084
GG	58	27.8	98	21.3	1.84(1.13–2.99)	0.014
-358 T > C						
TT	29	13.9	116	25.2		
CT	114	54.5	233	50.7	2.00(1.26–3.19)	0.004
CC	66	31.6	111	24.1	2.44(1.46–4.06)	0.001

^a^Adjusted for age, gender, and smoking status

**Table 3:** Genotype frequencies of *TNFSF15* polymorphisms and their association with NSCLC

Genotype	Patients(n = 340)		Controls(n = 460)		*OR* (95%*CI*)^a^	*P* value
	No	(%)	No	(%)		
-638 A > G						
AA	90	26.5	122	26.5		
AG	171	50.3	240	52.2	0.97(0.69–1.36)	0.867
GG	79	23.2	98	21.3	1.11(0.74–1.67)	0.605
-358 T > C						
TT	71	20.9	116	25.2		
CT	173	50.9	233	50.7	1.24(0.86–1.76)	0.248
CC	96	28.2	111	24.1	1.45(0.96–2.11)	0.074

^a^Adjusted for age, gender, and smoking status

### Stratification analysis of the *TNFSF15* polymorphism and the risk of SCLC

To evaluate the effect of smoking status and non-modifiable risk factors (age and gender) on the association of *TNFSF15*–638A > G with the risk of SCLC, we performed stratification analysis (Table 4). When stratified by gender, the *TNFSF15*–638GG genotype was associated with an increased risk of SCLC among males compared with the AA genotype (*OR* = 1.95, 95%*CI* = 1.09–3.45, *P* = 0.023), but not among females. Our data also showed that the risk of SCLC was associated with the −638GG genotype among older subjects with an *OR* (95% *CI*) of 2.93 (1.44–8.68), but not among younger subjects with an *OR* (95% *CI*) of 1.28 (0.71–2.34). However, no effect of the *TNFSF15*–638A > G variation on the risk of SCLC was found in stratified analysis based on smoking status.

**Table 4:** Association of *TNFSF15–*638A > G polymorphism with SCLC risk stratified by selected variables

Variables	Genotypes (Cases/Controls)			GG/AA model *OR* (95% *CI*)	*P* value
	GG	AG	AA		
Gender					
Male	42/72	84/170	28/94	1.95(1.09–3.45)	0.023
Female	16/26	28/70	11/28	1.41(0.54–3.70)	0.483
Age					
<60	37/62	71/149	30/66	1.28(0.71–2.34)	0.407
≥ 60	21/36	41/91	9/56	2.93(1.44–8.68)	0.006
Smoking status					
Non-smoker	28/49	53/124	18/60	1.79(0.88–3.65)	0.107
Smoker	30/49	59/116	21/62	1.70(0.86–3.38)	0.130

We then performed stratification analysis to evaluate the association of *TNFSF15* -358 T > C genotypes with SCLC (Table 5). Compared with the TT genotype, the CC genotype was associated with a higher risk of SCLC among females (*OR* = 8.42, 95%*CI* = 2.22–31.89, *P* = 0.002), but not among males. Age stratification analysis showed that there was a correlation between the CC genotype and the risk of SCLC (*OR* = 11.04, 95%*CI* = 3.57–34.15, *P* = 0.000) among elders when compared to TT carriers, but not among youngers. In addition, the CC genotype increased the risk of lung cancer among non-smokers (*OR* = 2.54, 95%*CI* = 1.20–5.35, *P* = 0.015) compared with the TT genotype.

**Table 5:** Association of *TNFSF15* -358 T > C polymorphism with SCLC risk stratified by selected variables

Variables	Genotypes (Cases/Controls)			CC/TT model *OR* (95% *CI*)	*P* value
	CC	CT	TT		
Gender					
Male	42/85	86/164	26/87	1.66(0.93–2.95)	0.086
Female	24/26	28/69	3/29	8.42(2.22–31.89)	0.002
Age					
<60	35/71	78/146	25/60	1.15(0.62–2.15)	0.653
≥ 60	31/40	36/87	4/56	11.04(3.57–34.15)	< 0.001
Smoking status					
Non-smoker	36/55	51/123	12/55	2.54(1.20–5.35)	0.015
Smoker	30/56	63/110	17/61	1.88(0.92–3.84)	0.086

## Discussion

Small-cell lung cancer (SCLC) is a deadly tumor with poor prognosis, which originates from high-grade malignant neuroendocrine cells [22]. Although sensitive to chemotherapy and radiotherapy, SCLC typically recurs rapidly after primary treatment and the five-year survival is only 6% after diagnosis [23]. Platinum-etoposide doublet has been officially approved for clinical use against SCLC [23]; however, few improvement has been made in SCLC treatment in past several years. Since SCLC is known as a stubborn cancer, there is an urgent need for the identification of biomarkers that can act as a potential therapeutic target in SCLC.

TNF superfamily members play an important role in cell proliferation, differentiation and apoptosis and are used for clinical treatment, or in clinical trials [24, 25]. TNFSF15, a member of *TNF* superfamily, is likely to inhibit the growth of tumors by suppressing neovascularization. TNFSF15 inhibits the proliferation of vascular endothelial cells in the G0 and G1 phases of cell cycle, and ultimately inhibits angiogenesis. *TNFSF15* gene encodes three splice variants, namely *VEGI*-174, *VEGI*-251 and *VEGI*-192 depending on the number of amino acids included [26]. *VEGI*-251, also known as *TL1A* (*TNF*-like molecule 1A), is the longest one of the splice variants. The combination of *TL1A* and *DR3* (Death Receptor 3) activates different signal transduction pathways by inducing *NF-kB* to activate initial T cell [10] and activating Caspases cascade to promote cell apoptosis [27]. These processes play an important role in the occurrence and development of tumors.

Till now, a few studies have been carried out to demonstrate the association of *TNFSF15* polymorphisms with the susceptibility to cancer. In our previous study, we found that *TNFSF15*–638A > G polymorphism was associated with the development of gastric adenocarcinoma [20]. In another study, authors indicated that *TNFSF15* rs6478106 is related to the risk of breast cancer in Chinese Han population [28]. In this study, we explored the association of *TNFSF15* variants with the susceptibility to lung cancer and found that -638A > G and -358 T > C variants elevated the risk of SCLC, but not of NSCLC. These findings suggested that the *TNFSF15* polymorphisms were involved in the development of various cancer types; however, the specific mechanism is not fully clear. We speculate that the TNFSF15 genetic variation affects the expression of TNFSF15 protein and then controls the downstream signal transduction molecules. These changes affect inflammatory and immune response, and further contribute to the development of cancers. Matijja and Clara found that an intron polymorphism of *TNFSF15* (rs6478108) affected the expression level of *TNFSF15* and increased NOD2-induced signaling and cytokines through caspase-8–induced IL-1 [29]. In our previous study, we found that the − 358 T > C polymorphism eliminates a nuclear factor Y (NF-Y) binding site and the -358C containing haplotypes had a significantly decreased reporter gene activity in gastric cells [20].

Tobacco smoking is recognized as one of the most important risk factors contributing to lung cancer [30]. Thus, we analyzed the effects of *TNFSF15* variants on SCLC by smoking status. Studies have shown a strong association between tobacco exposure and the development of SCLC [31, 32]. Cigarette smoke contains many carcinogenic chemicals such as nicotine and carbon monoxide tar. The complexity of cigarette smoke makes the mechanisms of developing lung cancer even more complicated. At least, tobacco smoking potentially alters the tumor immune microenvironment by creating DNA damage and causing inflammatory response [33, 34]. TNFSF15 is closely related to the inflammatory response. It has been reported that TNFSF15 can directly induce proinflammatory cytokines [35]. Long-term exposure of DNA to the carcinogens in tobacco smoke will lead to a higher mutation load in SCLC [36]. Our present data showed that *TNFSF15* -358 T > C polymorphism was related to SCLC among non-smokers instead of smokers, which needs more studies to explain. After stratified by smoking status, the sample size is not enough to evaluate the risk of this genetic variant with the risk of SCLC.

Age and gender are considered to be risk factors for tumor development and progression [37]. Our study showed the *TNFSF15*–638GG genotype elevated the risk of SCLC among males and individuals aged 60 years and older. Whether gender is related to the risk of lung cancer is controversial after taking into account smoking [38–40]. Due to the small size of several subgroups, a further larger-scale study needs to be conducted in order to carefully evaluate these findings.

In the future, it is necessary to evaluate the usability of these polymorphisms as a low-cost NSCLC screening tool for predicting individual lung cancer risk.

## Conclusion

Taken together, our results indicated that *TNFSF15* promoter polymorphisms might be involved in the development of SCLC.

## Abbreviations

NSCLC: non-small cell lung cancer
SCLC: small cell lung cancer
TNFSF15: Tumor necrosis factor superfamily member 15
